# Multiple intrinsic factors act in concert with Lhx2 to direct retinal gliogenesis

**DOI:** 10.1038/srep32757

**Published:** 2016-09-08

**Authors:** Jimmy de Melo, Brian S. Clark, Seth Blackshaw

**Affiliations:** 1 Johns Hopkins University School of Medicine, Solomon H. Snyder Department of Neuroscience, Baltimore, 21205, USA; 2 Johns Hopkins University School of Medicine, Department of Ophthalmology, Baltimore, 21205, USA; 3 Johns Hopkins University School of Medicine, Department of Neurology, Baltimore, 21205, USA; 4 Johns Hopkins University School of Medicine, Center for Human Systems Biology, Baltimore, 21205, USA; 5 Johns Hopkins University School of Medicine, Institute for Cell Engineering, Baltimore, 21205, USA

## Abstract

Müller glia (MG) are the principal glial cell type in the vertebrate retina. Recent work has identified the LIM homeodomain factor encoding gene *Lhx2* as necessary for both Notch signaling and MG differentiation in late-stage retinal progenitor cells (RPCs). However, the extent to which *Lhx2* interacts with other intrinsic regulators of MG differentiation is unclear. We investigated this question by investigating the effects of overexpression of multiple transcriptional regulators that are either known or hypothesized to control MG formation, in both wildtype and *Lhx2*-deficient RPCs. We observe that constitutively elevated Notch signaling, induced by *N1ICD* electroporation, inhibited gliogenesis in wildtype animals, but rescued MG development in *Lhx2*-deficient retinas. Electroporation of *Nfia* promoted the formation of cells with MG-like radial morphology, but did not drive expression of MG molecular markers. *Plagl1* and *Sox9* did not induce gliogenesis in wildtype animals, but nonetheless activated expression of the Müller marker P27^Kip1^ in *Lhx2*-deficient cells. Finally, *Sox2, Sox8*, and *Sox9* promoted amacrine cell formation in *Lhx2*-deficient cells, but not in wildtype retinas. These findings demonstrate that overexpression of individual gliogenic factors typically regulates only a subset of characteristic MG markers, and that these effects are differentially modulated by *Lhx2*.

Müller glia (MG) are adult radial glial cells that function as the primary physiological support cells within the retina. MG are specified from the progenitor cells of the retinal neuroepithelium (RPCs), which also generate retinal neurons, and share many morphological and molecular features with RPCs^1^,^2^,^3^. Both cell types feature radial processes that delimit the apical and basal surfaces of the retina^2^,^4^ and both co-express many transcription factors, neurofilament proteins, and signaling pathway molecules^3^,^5^,^6^. Furthermore, in teleost fish, MG are capable of functioning as adult stem cells following injury where they activate RPC-specific genes, undergoe proliferative expansion and then differentiate into retinal neurons to efficiently regenerate missing cells^7^,^8^,^9^,^10^. In contrast, mammalian MG are essentially quiescent, showing very limited regenerative capacity in experimental injury models^11^,^12^,^13^,^14^,^15^.

The identification of transcription factors (TFs) that selectively control MG specification and drive MG differentiation has been a considerable challenge, largely due to the overlap of gene expression between RPCs and MG. Several studies show that TFs in the retina that control gliogenesis are often also required for RPC maintenance, survival, proliferation, and/or progression through stages of developmental competence^16^,^17^,^18^,^19^,^20^,^21^. This shared gene expression also makes discrimination between immature MG precursors and RPCs difficult. This is particularly problematic when analysis is performed at late embryonic or early postnatal timepoints, when both RPCs and MG precursors co-exist.

Classic studies of retinal cell lineage used morphological criteria to identify MG^1^,^22^,^23^,^24^. Several MG-specific molecular markers have also been identified, including GLUL, P27^Kip1^, and RLBP1^25^,^26^,^27^. However, it is rare for both morphological criteria and multiple molecular markers to be tested for studies of any individual gene. It is also often unclear whether genes previously implicated as directing Müller gliogenesis are in fact truly sufficient to fully promote gliogenesis, or are required primarily for terminal differentiation and survival of MG. In this study we have used *in vivo* electroporation in neonatal mouse retina to misexpress genes encoding TFs implicated in MG differentiation in previous loss of function studies (*Sox2, Sox8, Sox9, Plagl1*)^28^,^29^,^30^, or whose expression has been detected in developing MG (*Nfia, Rax*)^31^,^16^.

Members of the Sry-related HMG-box (Sox) family are expressed in retinal progenitors and mature MG^28^,^29^. Inactivation of *Sox2, Sox8*, and *Sox9* disrupt differentiation and survival of MG^28^,^29^, but the ability of *Sox* family members to specify MG remains unclear. *Plagl1*, previously named *Zac1*, encodes a C2H2 zinc finger transcription factor. *Plagl1* has been shown to influence retinal cell fate decisions in *Xenopus,* with misexpression of *Plagl1* promoting MG development^30^. The functional role of *Plagl1* in the mammalian retina has not previously been characterized. The paired type homeodomain transcription factor *Rax* is expressed in developing MG, and retroviral transduction of RPCs with *Rax* induces expression of a subset of MG markers^16^. *Nfia* (Nuclear Factor I/A) was previously reported to be both necessary and sufficient to drive astrogliogenesis in cortex and spinal cord^32^,^33^. Intriguingly, *Nfia* expression is regulated by *Lhx2* in the developing hippocampus^34^. We have previously shown *Lhx2* to be an essential regulator of MG development^21^, but the role of *Nfia* during MG development has not previously been described.

We also analyzed the intracellular domain (ICD) of *Notch1*. Following ligand binding, the Notch intracellular domain (NICD) is cleaved and transported to the nucleus, where it forms an active transcriptional regulatory complex with the DNA-binding protein RBPJ and the co-activator MAML, thereby mediating its effects through regulation of target gene expression^35^. Early studies suggested that misexpression of Notch in the retina promoted Müller gliogenesis^16^. More recent analysis indicates that N1ICD is less directly instructive, instead functioning to maintain undifferentiated RPCs in a slowly proliferative state while blocking activation of neurogenic genes^36^,^37^.

In this report we assay whether these factors are sufficient to promote MG differentiation by analyzing expression of multiple MG specific markers, as well as cell morphology. Furthermore, we tested whether overexpression of these factors was sufficient to rescue Müller gliogenesis in cells lacking the LIM homeodomain TF *Lhx2*. We previously showed *Lhx2* to both be essential for Müller gliogenesis and to act as a direct global regulator of expression of multiple gliogenic factors and MG-specific genes^21^. We show that none of the tested factors were sufficient to promote all aspects of MG differentiation when overexpressed. Several were sufficient to promote the formation of cells with MG-like radial morphology or to activate expression of P27^Kip1^, a marker of cellular quiescence in MG^26^. However, none could activate expression of GLUL, a selective marker of differentiated MG^25^, in wildtype tissue. Furthermore, none of the electroporated TFs could fully rescue MG development following *Lhx2* loss of function. These results underscore the fact that few factors that are necessary for retinal gliogenesis are also sufficient to induce glial differentiation, and highlights the central role of *Lhx2* in organizing and coordinating MG differentiation.

## Results

### Misexpression of *N1ICD* in the mammalian retina promotes RPC maintenance, and is sufficient to rescue MG development following loss of *Lhx2* expression

**Figure 1:**
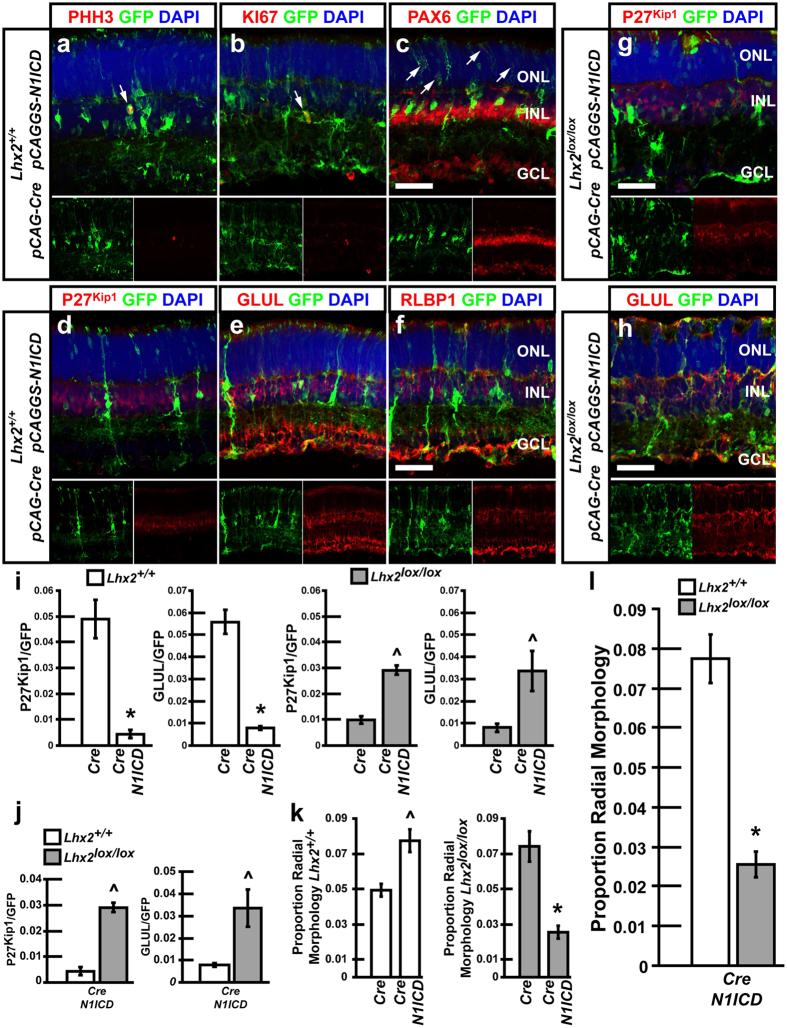
Electroporation of *N1ICD* maintains radial RPCs and is sufficient to rescue loss of MG development resulting from *Lhx2* loss of function. (**a–f**) *Lhx2*^+/+^ retinas electroporated with *Cre/GFP/N1ICD*. (**a–c**) Fluorescent immunohistochemical labeling of electroporated retinas with GFP and the proliferation markers PHH3 and KI67, or the progenitor/amacrine/ganglion cell marker PAX6. Arrows indicate co-labeled cells. (**d–f**) fluorescent co-labeling with the MG markers P27^Kip1^, GLUL, and RLBP1. (**g,h**) *Lhx2*^*lox/lox*^ retinas electroporated with *Cre/GFP/N1ICD* and analyzed by fluorescent immunohistochemical labeling with P27^Kip1^ and GLUL. (**i,j**) Quantification of GFP/P27^Kip1^ and GFP/GLUL co-labeled cells in *Lhx2*^+/+^ and *Lhx2*^*lox/lox*^ mice following *Cre/GFP* or *Cre/GFP/N1ICD*. (**k,l**) Quantification of radial cells in *Lhx2*^+/+^ or *Lhx2*^*lox/lox*^ mice following *Cre/GFP* or *Cre/GFP/N1ICD* electroporation. *Indicates significant decrease while ^ indicates significant increases (P < 0.05, N = 6 for marker counts, N = 12 for radial morphology counts). ONL, outer nuclear layer; INL inner nuclear layer; GCL, ganglion cell layer. Scale bars: 50 μm (**c,f,g,h**).

We electroporated neonatal mouse retinas with control *pCAG-Cre/pCALNL-GFP (Cre*) or *pCAGGS-N1ICD/pCAG-Cre/pCALNL-GFP (N1ICD/Cre*) constructs and analyzed the electroporated retinas at postnatal day (P)14. The *pCAG-Cre* plasmid constitutively expresses Cre recombinase, while *pCALNL-GFP* expresses the GFP fluorescent reporter following Cre mediated excision of a transcriptional stop site. The *pCAGGS-N1ICD* plasmid constitutively expresses the N1ICD domain. In wildtype (WT) retinas electroporated with *Cre*, we observed that approximately 5% of electroporated cells expressed the MG markers P27^Kip1^, GLUL, or displayed radial morphology characteristic of MG, where cell processes extended from the basal inner limiting membrane to the apical outer limiting membrane (4.9% P27^Kip1^ +ve; 5.6% GLUL +ve; 4.9% MG-like radial morphology) (Fig. 1a–f,i,k). Electroporation of *N1ICD* dramatically reduced the number of P27^Kip1^ and GLUL-expressing cells to 0.4 and 0.7% respectively (Fig. 1d,e,i). Furthermore, co-labeling with the MG marker RLBP1 was not detected (Fig. 1f). Conversely, the proportion of cells exhibiting radial morphology significantly increased to 7.7% (Fig. 1k).

The generation of cells with radial morphology, but not MG marker expression, suggested that electroporation of *N1ICD/Cre* promoted the maintenance of undifferentiated radial RPCs at P14, validating previous reports^36^. We immunostained *N1ICD/Cre* electroporated retinas for two markers of actively proliferating cells, Phosphohistone H3 (PHH3) and KI67, as well as the RPC-expressed transcription factor PAX6. We found that subsets of electroporated cells were labeled with both PHH3 and KI67, whereas co-labeling was never observed in *Cre* controls (Fig. 1a,b). Furthermore, ectopic co-labeling of PAX6 was detected in the outer nuclear layer (ONL) of the retina, where PAX6 is not normally expressed at P14 (Fig. 1c).

We previously reported that *Lhx2*, which encodes a LIM homeodomain transcription factor, is necessary for MG development^21^. *Lhx2* drives MG development in part by directly activating expression of Notch signaling pathway genes^21^. Here we electroporated *Cre* or *N1ICD/Cre* into *Lhx2*^*lox/lox*^ retinas to determine whether *N1ICD* could rescue the loss of MG resulting from *Lhx2* knockout. Electroporation of *Cre* into *Lhx2*^*lox/lox*^ retinas resulted in a dramatically reduced proportion of P27^Kip1^ and GLUL-labeled MG, as reported previously (Fig. 1i)^21^. Interestingly, we observed an increase in the number of radial cells following electroporation of *Cre* into *Lhx2*^*lox/lox*^ retinas (Fig. 1k). We previously showed that *Lhx2* loss of function does not result in an increase of proliferating RPCs^21^. Electroporation of *N1ICD/Cre* into *Lhx2*^*lox/lox*^ rescued the number of cells expressing both P27^Kip1^ and GLUL, while significantly reducing the number of cells featuring MG-like radial morphology (Fig. 1g–l). The proportion of cells expressing either MG markers or showing radial morphology was similar (P27^Kip1^ +ve 2.9%, GLUL +ve 3.4%, radial morphology 2.6%). The number of cells expressing MG markers was comparable to controls, where *Cre* was electroporated into WT retinas, though the number of radial cells remained statistically reduced (p = 0.05, N = 6 each condition, 12 total eyes, P27^Kip1^; P > 0.05, N = 6 each condition, 12 total eyes, GLUL; P < 0.05, N = 12 each condition, 24 total eyes, MG-like radial morphology) (Fig. 1i,k).

### *Nfia* promotes incomplete MG development, and partially rescues MG differentiation following *Lhx2* loss of function

**Figure 2:**
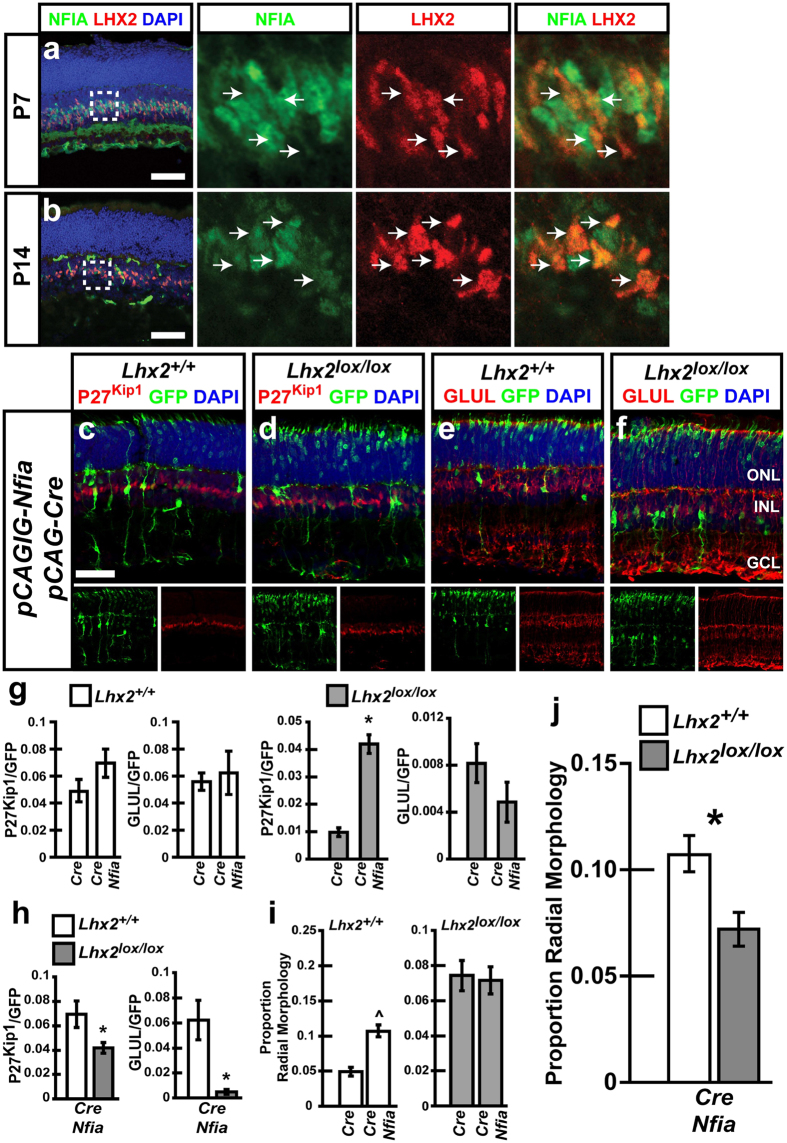
NFIA is expressed in retinal MG and electroporation of *Nfia* promotes the formation of radial cells and is sufficient to rescue loss of P27^Kip1^ expression resulting from *Lhx2* loss of function. (**a,b**) Immunohistochemical co-labeling of NFIA with LHX2 at P7 and P14, arrows indicate co-labeled cells. (**c–f**) Electroporation of *Lhx2*^+/+^ and *Lhx2*^*lox/lox*^ retinas with *Cre/GFP/Nfia* and analyzed by immunohistochemical co-labeling of GFP with the MG markers P27^Kip1^ and GLUL. (**g,h**) Quantification of GFP/P27^Kip1^ and GFP/GLUL co-labeled cells in *Lhx2*^+/+^ and *Lhx2*^*lox/lox*^ mice following *Cre/GFP* or *Cre/GFP/Nfia*. (**i,j**) Quantification of radial cells in *Lhx2*^+/+^ or *Lhx2*^*lox/lox*^ mice following *Cre/GFP* or *Cre/GFP/Nfia* electroporation. *Indicates significant decrease (P < 0.05, N = 6 for marker counts, N = 12 for radial morphology counts). Scale bars: 50 um (**a–c**).

The retinal localization and function of *Nfia* has not previously been established. We utilized a monoclonal antibody specific for NFIA to assess retinal expression and localization. NFIA protein expression became restricted to the medial inner nuclear layer (INL) of the retina by P7 and co-localized with LHX2 in MG (Fig. 2a). NFIA +ve cells that did not co-express LHX2 were also detected at P7. At P14, NFIA co-localized with LHX2 in MG with relatively fewer NFIA +ve/LHX2 − ve cells present (Fig. 2b). We did not detect any LHX2 +ve cells that did not co-label with NFIA at either timepoint, indicating that all MG express NFIA.

We next electroporated *pCAG-Nfia/pCAG-Cre/pCALNL-GFP (Nfia/Cre*) into neonatal retinas, and assessed MG marker expression at P14 (Fig. 2c,e). The *pCAG-Nfia* plasmid constitutively expresses NFIA. Electroporation of *Nfia/Cre* did not significantly alter the proportion of P27^Kip1^ or GLUL expressing cells in the retina as compared to *Cre* in control experiments, though non-significant increases were observed for both markers (Fig. 2g). Electroporation of *Nfia/Cre* resulted in a significant increase in the proportion of MG-like radial cells, with twice as many detected compared to *Cre* controls (Fig. 2i). Together, these results suggest that misexpression of *Nfia/Cre* may be sufficient to promote morphological, but not molecular, characteristics of MG in WT retina.

Previous studies observed that endogenous expression of *Lhx2* in hippocampal progenitor cells may override the gliogenic activity of *Nfia*, and that misexpression of *Nfia* with concurrent *Lhx2* loss of function promoted hippocampal astrogliogenesis^34^. We tested whether *Nfia* was sufficient to promote MG development with *Lhx2* loss of function by electroporating *Nfia/Cre* into *Lhx2*^*lox/lox*^ mice. *Nfia/Cre* was sufficient to rescue the proportion of P27^Kip1^ +ve cells (Fig. 2g). The proportion of cells expressing GLUL was unchanged from that observed following electroporation of *Cre* into *Lhx2*^*lox/lox*^ mice, and remained significantly reduced compared to wild type animals (Fig. 2g). The proportion of MG-like radial cells seen in *Lhx2*^*lox/lox*^ mice electroporated with *Nfia/Cre* was unchanged from *Lhx2*^*lox/lox*^ mice electroporated with *Cre* (Fig. 2i). Furthermore, the proportion of radial cells generated in *Lhx2*^*lox/lox*^ retinas represented a significant decrease from levels seen following electroporation of *Nfia/Cre* into wild type animals (Fig. 2j).

### *Rax* and *Plagl1* are not sufficient to promote MG development, but *Plagl1* partially rescues MG development following *Lhx2* loss of function

**Figure 3:**
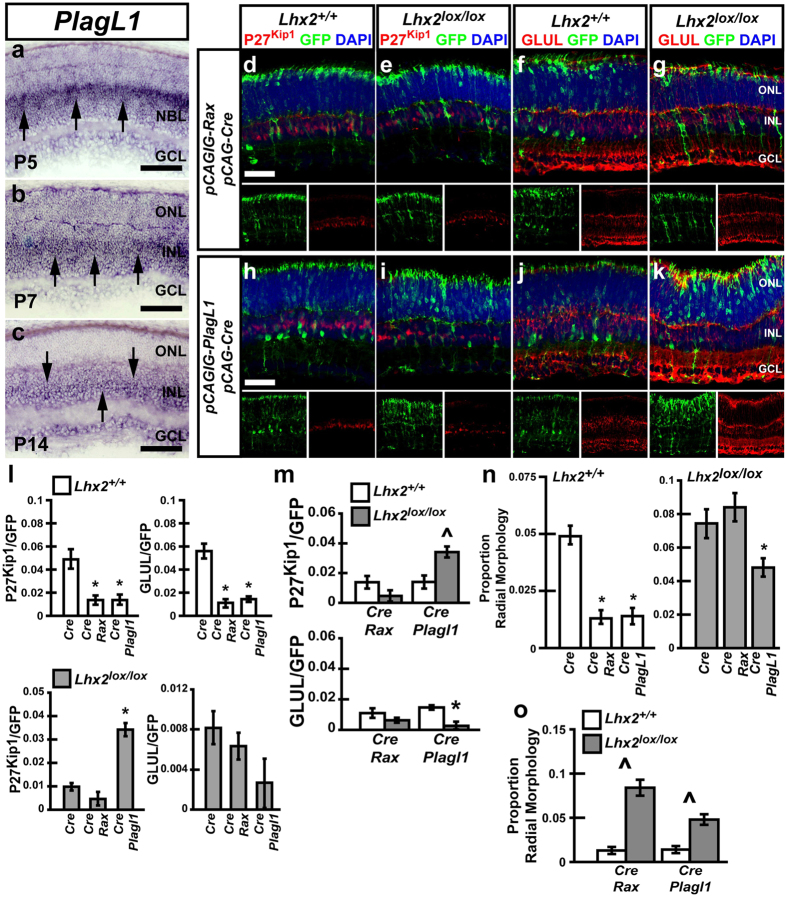
Electroporation of *Rax* both blocks MG development and fails to rescue the loss of MG resulting from *Lhx2* loss of function, whereas *Plagl1* blocks MG development but is sufficient to rescue P27^Kip1^ expression resulting from *Lhx2* loss of function. (**a–c**) *In situ* hybridization of *Plagl1* at P5, P7, and P14. Arrows show regions of *Plagl1* RNA enrichment. (**d–g**) Electroporation of *Lhx2*^+/+^ and *Lhx2*^*lox/lox*^ retinas with *Cre/GFP/Rax* and analyzed by immunohistochemical co-labeling of GFP with the MG markers P27^Kip1^ and GLUL. (**h–k**) Electroporation of *Lhx2*^+/+^ and *Lhx2*^*lox/lox*^ retinas with *Cre/GFP/Plagl1* and analyzed by immunohistochemical co-labeling of GFP with P27^Kip1^ and GLUL. (**l,m**) Quantification of GFP/P27^Kip1^ and GFP/GLUL co-labeled cells in *Lhx2*^+/+^ and *Lhx2*^*lox/lox*^ mice following *Cre/GFP, Cre/GFP/Rax or Cre/GFP/PlagL1* electroporation. (**n,o**) Quantification of radial cells in *Lhx2*^+/+^ or *Lhx2*^*lox/lox*^ mice following *Cre/GFP, Cre/GFP/Rax or Cre/GFP/PlagL1* electroporation. *Indicates significant decrease while ^ indicates significant increases (P < 0.05, N = 6 for marker counts, N = 12 for radial morphology counts). NBL, neuroblastic layer. Scale bars: 100 μm (**a–c**), 50 μm (**d,h**).

The retinal expression pattern of *Plagl1* in the mouse has not been reported. We performed *in situ* hybridization to detect *Plagl1* RNA expression in the retina, and found expression consistent with MG localization (Fig. 3a–c). RNA expression was identified in the medial neuroblastic layer (NBL) at P5 (Fig. 3a), consistent with the location of differentiating MG. *Plagl1* was similarly enriched in the medial INL, where MG are located at P7 (Fig. 3b). By P14, *Plagl1* expression is less clearly concentrated in the medial INL, although expression remains generally enriched in the INL (Fig. 3c). Cumulatively, these results indicate that *Plagl1* expression is enriched in developing MG.

To determine whether *Rax* or *Plagl1* were sufficient promote MG development we electroporated neonatal mice with either *pCAG-Rax/pCAG-Cre/pCALNL-GFP (Rax/Cre*) or *pCAG-Plagl1/pCAG-Cre/pCALNL-GFP (Plagl1/Cre*), and assayed for MG formation. The *pCAG-Rax* plasmid constitutively expresses RAX, while *pCAG-Plagl1* constitutively expresses PLAGL1. Contradicting previous reports, misexpression of *Rax* into WT mice resulted in significant decreases in expression of the MG markers P27 ^Kip1^ and GLUL, as well as decreases in MG-like radial cells (Fig. 3d,f,l,n). Electroporation of *Rax/Cre* also notably altered the position of rod photoreceptors, with photoreceptor soma concentrating near the apical ONL adjacent to the retinal outer limiting membrane, instead of positioning randomly throughout the ONL (Fig. 3d,f). Similarly, WT retinas electroporated with *Plagl1/Cre* showed significantly reduced expression of both MG markers (Fig. 3h,j,l), as well as reduced numbers of MG-like radial cells (Fig. 3n).

Though neither factor proved sufficient to promote MG development, we tested whether either *Rax/Cre* or *Plagl1/Cre* could rescue the loss of MG development seen following *Lhx2* loss of function. Electroporation of *Rax/*Cre into neonatal *Lhx2*^*lox/lox*^ retinas resulted in no significant change in the number of cells expressing either P27 ^Kip1^ or GLUL (Fig. 3e,g,l) as compared to *Cre* controls. Interestingly, the proportion of MG generated following electroporation of *Rax/Cre* into WT and *Lhx2*^*lox/lox*^ retinas does not significantly differ, indicating that misexpression of *Rax* blocked MG development to a similar extent as loss of *Lhx2* function. The number of cells featuring MG-like radial morphology was also unchanged following electroporation of *Rax/Cre* into *Lhx2*^*lox/lox*^ mice compared to electroporation of *Cre*.

Electroporation of *Plagl1/Cre* into *Lhx2*^*lox/lox*^ retinas, however, resulted in a significant rescue of expression of P27 ^Kip1^ but not GLUL (Fig. 3i,k,l). Despite increased P27 ^Kip1^ expression, the number of MG-like radial cells generated was reduced compared to *Lhx2*^*lox/lox*^ retinas electroporated with *Cre* (Fig. 3n). Cumulatively, our results indicate that neither *Rax* nor *Plagl1* is sufficient to promote MG development in the mouse retina. *Plagl1* however may be functionally redundant for *Lhx2* in the activation of P27^Kip1^.

### Sry-related HMG-box (Sox) family members *Sox2, Sox8, Sox9* are insufficient to promote MG development, but drive amacrine cell differentiation in the absence of *Lhx2*

**Figure 4:**
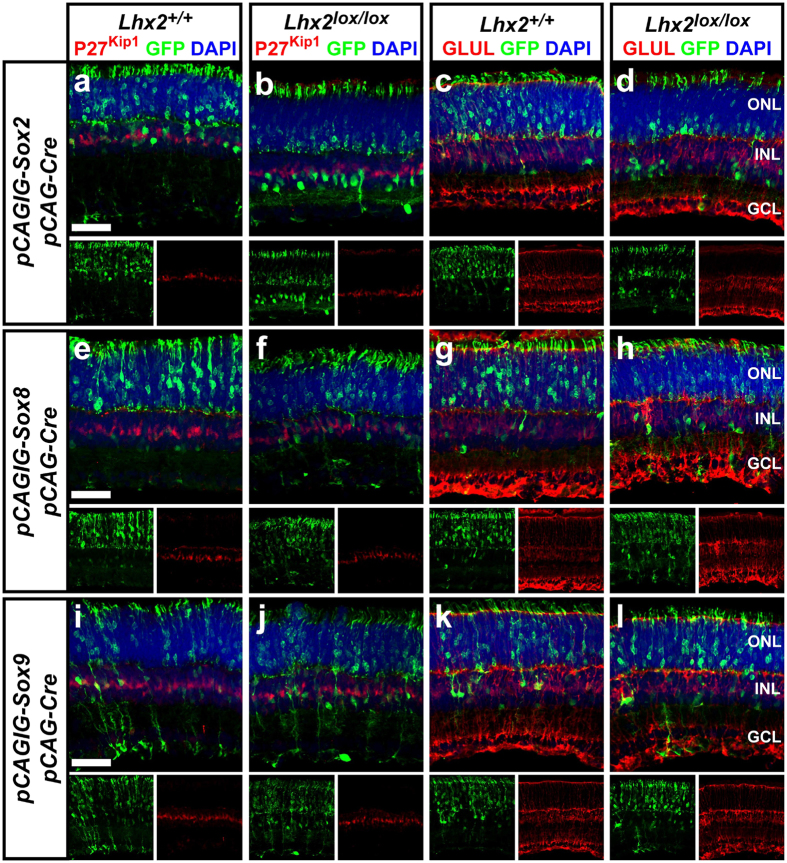
Electroporation of *Sox2, Sox8* and *Sox9* in the mouse retina. (**a–d**) Electroporation of *Lhx2*^+/+^ and *Lhx2*^*lox/lox*^ retinas with *Cre/GFP/Sox2* and analyzed by immunohistochemical co-labeling of GFP with the MG markers P27^Kip1^ and GLUL. (**e–h**) Electroporation of *Lhx2*^+/+^ and *Lhx2*^*lox/lox*^ retinas with *Cre/GFP/Sox8* and analyzed by immunohistochemical co-labeling of GFP with the MG markers P27^Kip1^ and GLUL. (**i–j**) Electroporation of *Lhx2*^+/+^ and *Lhx2*^*lox/lox*^ retinas with *Cre/GFP/Sox9* and analyzed by immunohistochemical co-labeling of GFP with the MG markers P27^Kip1^ and GLUL. Scale bars: 50 um (**a,e,i**).

**Figure 5:**
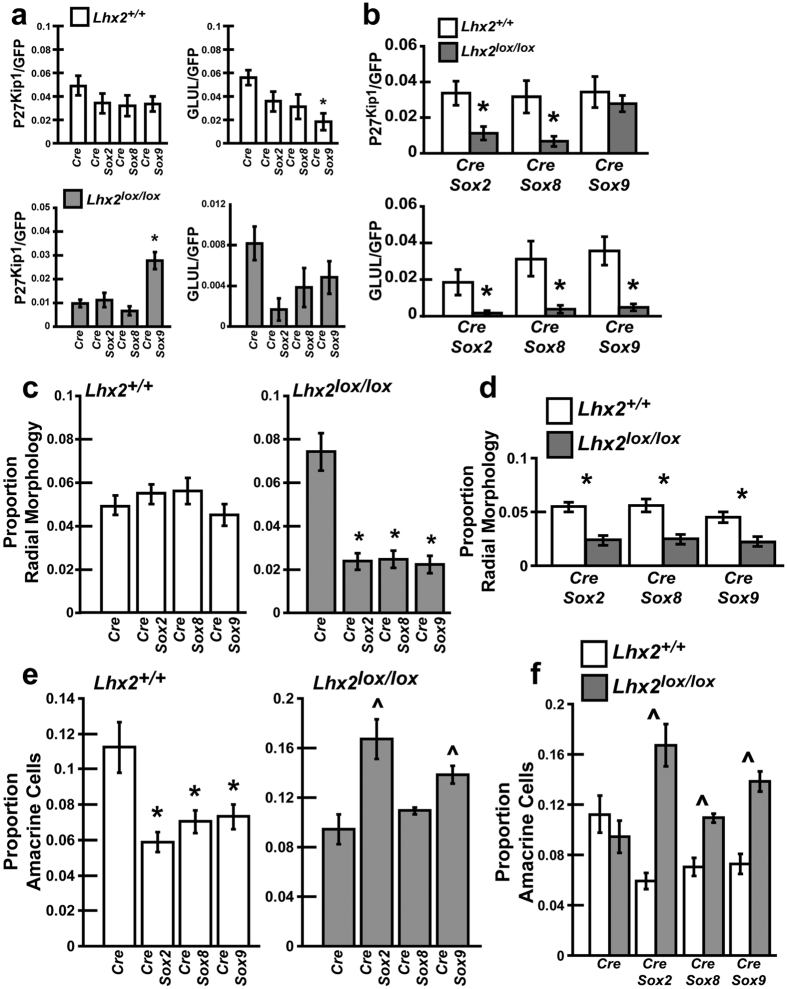
Electroporation of *Sox2, Sox8,* or *Sox9* are insufficient to promote MG development and largely fail to rescue the loss of MG resulting from *Lhx2* loss of function, instead promoting amacrine cell development. (**a,b**) Quantification of GFP/P27^Kip1^ and GFP/GLUL co-labeled cells in *Lhx2*^+/+^ and *Lhx2*^*lox/lox*^ mice following *Cre/GFP, Cre/GFP/Sox2, Cre/GFP/Sox8, or Cre/GFP/Sox9* electroporation. (**c,d**) Quantification of radial cells in *Lhx2*^+/+^ or *Lhx2*^*lox/lox*^ mice following *Cre/GFP, Cre/GFP/Sox2, Cre/GFP/Sox8, or Cre/GFP/Sox9* electroporation. (**e,f**) Quantification of amacrine cells by morphology in *Lhx2*^+/+^ or *Lhx2*^*lox/lox*^ mice following *Cre/GFP, Cre/GFP/Sox2, Cre/GFP/Sox8, or Cre/GFP/Sox9* electroporation. *Indicates significant decrease while ^ indicates significant increases (P < 0.05, N = 6 for marker counts and amacrine cell counts, N = 12 for radial morphology counts).

To test the sufficiency of *Sox2, Sox8*, and *Sox9* to promote MG development we electroporated neonatal WT retinas with *pCAG-Sox2/pCAG-Cre/pCALNL-GFP, pCAG-Sox8/pCAG-Cre/pCALNL-GFP*, or *pCAG-Sox9/pCAG-Cre/pCALNL-GFP*, herein referred to as *Sox2/Cre, Sox8/Cre*, or *Sox9/Cre*, and assayed for MG. The *pCAG-Sox2, -Sox8, -Sox9* plasmids constitutively expresses SOX2, SOX8, and SOX9 respectively. Electroporation of *Sox2/Cre* or *Sox8/Cre* did not result in significant changes in the number of MG as determined by marker expression or MG-like radial morphology at P14 (Figs 4a,c,e,g and 5a,c). Electroporation of *Sox9/Cre* also did not affect expression of P27^Kip1^ or the proportion of radial cells, but did result in significantly decreased GLUL expression (Figs 4i,k and 5a,c).

We next tested whether electroporation of *Sox* genes could rescue the loss of MG resulting from *Lhx2* loss of function. We electroporated *Lhx2*^*lox/lox*^ mice with *Sox2/Cre, Sox8/Cre*, or *Sox9/Cre* and quantified the numbers of MG generated at P14. *Sox2* and *Sox8* were insufficient to rescue MG marker expression, with the proportion of P27^Kip1^ or GLUL expressing cells identical to that of *Lhx2*^*lox/lox*^ animals electroporated with *Cre* (Figs 4b,d,f,h and 5a,b). *Sox9* did rescue P27^Kip1^ expression, but not GLUL expression (Figs 4j,l and 5a,b). The proportion of P27^Kip1^ labeled cells was not significantly different from that of WT mice electroporated with *Cre* or *Cre/Sox9*. Interestingly, the number of MG-like radial cells was significantly reduced following electroporation of *Cre/Sox2, Cre/Sox8*, and *Cre/Sox9* into *Lhx2*^*lox/lox*^ animals, compared with *Lhx2*^*lox/lox*^ animals electroporated with *Cre* (Figs 4b,d,f,h,j,l and 5c). The number of MG-like radial cells generated was also significantly reduced compared to WT mice electroporated with the respective *Sox* genes (Fig. 5d).

The loss of radial cells in *Lhx2*^*lox/lox*^ mice was coupled with an increase of cells localized in the basal INL, consistent with amacrine cells. To characterize the role of *Sox* genes in the regulation of amacrine cell development we electroporated WT and *Lhx2*^*lox/lox*^ mice and scored the number of amacrine cells generated. Electroporation of *Sox2/Cre, Sox8/Cre*, or *Sox9/Cre* in WT mice yielded significantly decreased numbers of amacrine cells as compared to *Cre* controls (Fig. 5e). Conversely, electroporation of *Sox2/Cre* and *Sox9/Cre* but not *Sox8/Cre* into *Lhx2*^*lox/lox*^ animals resulted in a notable increase in amacrine cells, compared to Cre electroporated controls (Fig. 5e). *Sox2/Cre, Sox8/Cre*, and *Sox9/Cre* electroporations all generated significantly more amacrine cells in *Lhx2*^*lox/lox*^ than WT animals (Fig. 5f). Cumulatively, these results suggest that *Sox2, Sox8*, and *Sox9* are insufficient to promote MG development, but all strongly promote amacrine cell development in the absence of *Lhx2* expression.

## Discussion

This study offers a reevaluation of the phenotypes seen following overexpression of retinal transcriptional regulators that have been previously implicated in MG development. Our observations corroborate many previously observed phenotypes, while qualifying and adding context to others. Overexpression of individual glial-enriched TFs had varying effects on MG generation and differentiation, and were likewise differentially regulated by *Lhx2*. These results are summarized in Table 1.

In general, overexpression of these factors in a WT background did not promote MG formation. Only *N1ICD* and *Nfia* overexpression lead to an increase in cells with MG-like radial morphology, but failed to induce expression of either P27^Kip1^ or GLUL, with *N1ICD* reducing expression of both markers. Indeed, *Rax* and *Plagl1* both significantly decreased the fraction of cells showing MG-like radial morphology and expressing MG markers. These results stand in sharp contrast to the potently gliogenic Notch target *Hes5*, which robustly drives expression of both markers^21^. Electroporation of *PlagL1, Sox2, Sox8*, and *Sox9* had no affect on the population density (cells/μm^2^) of surviving electroporated cells at P14 (Fig. S1). Electroporation of *N1ICD* and *Nfia* resulted in a general reduction of the number of electroporated cells/μm^2^ while *Rax* resulted in an increase in cells/μm^2^ (Fig. S1). Overexpression of several different individual factors in *Lhx2*-deficient cells lead to rescued expression of P27^Kip1^, but only *N1ICD* was able to rescue expression of GLUL. Finally, we uncover a previously unknown role for *Lhx2* in suppressing Sox-dependent formation of amacrine cells. These findings highlight the complex combinatorial molecular relationships controlling MG differentiation.

In the specific case of *N1ICD*, constitutive Notch signaling induced electroporated cells to adopt a RPC-like state, with increased numbers of proliferative radial cells displaying decreased MG marker expression. This stands in sharp contrast to overexpression of the Notch pathway effector *Hes5*, which induced dramatic increases in the fraction of cells with MG-like radial morphology (*Cre* = 0.049 ± .0011; *Hes5/Cre* = 0.182 ± .0040, n = 12.), as well as P27^Kip1^ and GLUL labeling^21^. Ectopic expression of the RPC marker PAX6, and the proliferation markers KI67 and PHH3, was observed following *N1ICD* electroporation. These results fit well with previous reports that indicate that ectopic activation of Notch in the retina promotes progenitor maintenance and blocks cell cycle exit and terminal differentiation^36^,^37^.

We have previously demonstrated that *Lhx2* function was required for Müller gliogenesis, and that MG development was disrupted in *Lhx2* knockouts^21^. *Lhx2* directly activates expression of Notch pathway genes in RPCs, consequently conditional inactivation of *Lhx2* results in rapid down-regulation of Notch signaling. Whether *Lhx2* mediated its effects on Müller development through direct regulation of MG gene expression, or indirectly via regulation of Notch pathway gene expression, was unclear. Interestingly, *Lhx2* also regulates RPC progression through successive competence states^38^. Loss of function of *Lhx2* leads to cell cycle exit, resulting in progenitor depletion and restricted neurogenesis, effects similar to loss of Notch function^37^,^38^,^39^. These studies suggest that *Lhx2* controls MG differentiation in large part by regulating Notch pathway activation. Indeed, rapid RPC dropout coupled with failed gliogenesis may account for the increased number of radial cells which lack expression of MG markers following *Lhx2* loss of function in this study.

Here, we demonstrate that misexpression of *N1ICD* was sufficient to rescue the effects of *Lhx2* loss of function. Expression of both P27^Kip1^ and GLUL were restored, although the proportion of cells expressing both remained slightly reduced compared to WT controls. The fraction of cells with MG-like radial morphology was also reduced, indicating *N1ICD* may rescue RPC dropout resulting from *Lhx2* loss of function. These data indicate that *Lhx2* promotes Müller gliogenesis largely through regulation of the Notch signaling pathway, and is at least partially dispensable for regulation of MG gene expression. Similar observations have been made regarding the role of *Sox2* in MG development, wherein ectopic activation of Notch signaling was sufficient to rescue MG development in *Sox2* knockouts^40^. Interestingly, we previously reported that overexpression of *Hes5*, a potently Müller gliogenic Notch transcriptional effector, was insufficient to rescue the effects of *Lhx2* loss of function, with the fraction of cells expressing P27^Kip1^, GLUL^21^ or displaying radial morphology (*Cre/Lhx2*^*lox/lox*^ = 0.074 ± .0029; *Hes5/Cre/Lhx2*^*lox/lox*^ = 0.087 ± .0026, n = 12), unchanged. Rescue of MG differentiation with *N1ICD* but not *Hes5* in *Lhx2*-deficient cells suggests that concurrent activation of multiple Notch pathway target genes is required for MG differentiation, and that the gliogenic function of individual Notch pathway effectors such as *Hes5* is instructive only in the context of functional Notch signaling.

*Nfia*, which strongly promotes astrogliogenesis elsewhere in the CNS, showed mixed effects on generation of MG, despite its strong and selective expression in late-stage RPCs and mature MG. Though electroporation of *Nfia* triggered a significant increase in the fraction of MG-like radial cells in WT retina, with twice the fraction of electroporated cells showing clear radial morphology, it did not induce any significant change in MG marker expression. Furthermore, no effect on the fraction of cells with MG-like radial morphology was seen following electroporation of *Nfia/Cre* into *Lhx2*^*lox/lox*^ mice. Despite not activating MG marker expression in WT mice, *Nfia* overexpression was sufficient to rescue P27^Kip1^ expression in *Lhx2* knockouts. The gliogenenic effects of *Nfia* have previously been shown to be *Lhx2*-dependent in the hippocampus, where *Nfia* misexpression blocks neurogenesis and promotes astrogliogenesis^34^. We see a similar phenomenon, wherein electroporation of *Nfia* into WT mice seemingly blocks neurogenesis in the retina, resulting in a proportional increase of MG-like radial cells. This increase in radial cells was similar to that seen following electroporation of *N1ICD,* as was the rescue of P27^Kip1^ expression in the *Lhx2* knockouts. *Nfia* can function as a downstream transcriptional effector of the Notch pathway^41^, and our data suggests that this remains true of *Nfia* in the retina. Rescue of expression of the Cdk inhibitor P27^Kip1^ indicates that *Nfia* may play a key role in regulating MG quiescence, a known role of Notch in MG^42^, and we speculate that Notch may regulate MG quiescence in part through *Nfia*.

We found that electroporation of both *Rax* and *Plagl1* potently inhibited Müller gliogenesis. Previous studies reported that retroviral transduction of neonatal rat retinas with *Rax* resulted in 90% of transduced cells displaying radial morphology and expressing MG markers such as CRALBP^16^, while injection of *Plagl1* into Xenopus embryos resulted in a 4-fold increase in MG^30^. In the case of *Plagl1*, the divergence in phenotypes observed may simply reflect evolutionary differences between amphibians and mammals in the transcriptional circuitry controlling gliogenesis. However, we did observe that electroporation of *Plagl1/Cre* in *Lhx2*^*lox/lox*^ mice did partially rescue P27^Kip1^ expression, though the proportion of P27^Kip1^expressing cells remained notably lower than that in WT controls. *Plagl1* may therefore contribute to MG quiescence in the mammalian retina.

While electroporation of *Rax* potently inhibited gliogenesis, it also led to observable photoreceptor phenotypes, where photoreceptor cell nuclei were shifted to the outer region of the outer nuclear layer. That *Rax* electroporation affects photoreceptor development is consistent with a previously demonstrated role of *Rax* in later stages of photoreceptor differentiation^43^. The complete lack of gliogenic effects resulting from *Rax* electroporation may also be due to high levels of *Rax* expression induced by electroporation of CAG-based expression plasmids in this study^44^,^45^, compared to the relatively weak retroviral promoters used to drive *Rax* expression in previous reports^16^. RAX competes directly with the Notch effectors HES1, HES5, and HEY1 for binding of the embryonic enhancer locus for photoreceptor *Otx2* transcription (EELPOT), with RAX activating *Otx2* and promoting photoreceptor specification via EELPOT while HES1, HES5, and HEY1 repress *Otx2*^46^. The blockade of MG development in our study may thus result from constitutive activation of *Otx2* expression following *Rax* electroporation.

One of the more surprising findings of this study was the general failure of *Sox* family transcription factors to promote MG development. *Sox2, Sox8*, and *Sox9* have all been shown to be required for the development of MG in the mammalian retina^28^,^29^. Previous work also indicated that *Sox8* and *Sox9* loss of function resulted in compensatory increases in the fraction of photoreceptors, complementing the reduction of MG^29^. That study did not observe any increase in the fraction of MG following overexpression of *Sox8* and *Sox9* in late embryonic RPCs. We also found that electroporation of *Sox* family members failed to promote Müller gliogenesis, and largely failed to rescue MG formation following *Lhx2* loss of function, although *Sox9* did restore P27^Kip1^ expression. Cumulatively, our data indicates that while *Sox2, Sox8* and *Sox9* may be necessary for MG development, none appears sufficient for retinal gliogenesis.

Interestingly, we found that *Lhx2* seemed to constrain the ability of *Sox2, Sox8* and *Sox9* to drive amacrine cell differentiation. *Sox2*-dependent promotion of amacrine specification was previously reported in E17 explants following retroviral overexpression^28^. *Sox2* has also been shown to be necessary for differentiation of individual AC subtypes^47^. We found that electroporation of *Sox2/Cre, Sox8/Cre*, and *Sox9/Cre* into *Lhx2*^*lox/lox*^ mice resulted in a substantially increased proportion of amacrine cells compared with electroporation of WT mice. Electroporation of *Sox2/Cre* and *Sox9/Cre* both significantly boosted amacrine cell numbers in *Lhx2*^*lox/lox*^ mice compared to electroporation of *Cre* alone. This suggests that in addition to being necessary for retinal gliogenesis, *Lhx2* may also play a more general role in modulating and constraining levels of neurogenesis in late-stage RPCs, as has been described in the cortex and hippocampus^34^,^48^,^49^. Whether this modulation is achieved indirectly through regulation of Notch or directly through competition and interaction at common target loci is unclear. Ectopic activation of Notch via misexpression of *N1ICD* rescued gliogenesis in Sox2 knockouts^40^, and similarly rescued gliogenesis resulting from *Lhx2* loss of function in this study. These observations indicate that Notch pathway regulation by *Lhx2* and *Sox* family members contributes significantly to controlling the balance of amacrine cell and MG generation.

## Methods

### Animals

Timed pregnant female CD-1 mice were purchased from Charles River Laboratories (Wilmington, MA) and pups of either sex were used for electroporation. *Lhx2*^*lox/lox*^ mice (obtained from Dr. Edwin Monuki, University of California, Irvine) have been described^50^. Timed pregnancies of *Lhx2*^*lox/lox*^ mice were initiated and pups of either sex were used for electroporation. All procedures were approved by and carried out in accordance with guidelines approved by the Institutional Animal Care and Use Committee at the Johns Hopkins University School of Medicine, in accordance with NIH guidelines (protocol #MO16M62).

### Cell counts

All counts were performed blindly on whole retinal sections as previously described^51^. The total number of cells counted for each condition is presented in Table S1. Differences between two means were assessed by unpaired two-tailed Student’s t-test. Radial MG morphology was scored by tracing GFP labeling of individual cells from the inner limiting membrane through the inner plexiform and nuclear layers of the retina, past the outer plexiform layer and into the outer nuclear layer. Amacrine cells were scored based on morphology visualized by GFP labeling using the following criteria: cell soma positioned in the inner (basal) inner nuclear layer, dendrite extension into the inner plexiform layer but not extending beyond the retinal ganglion cell layer, and absence of an apical process extending to the outer plexiform layer. The number of electroporated cells/μm^2^ was determined for each condition in WT animals by counting GFP labeled cells and dividing by the area of the confocal imaging field (49506.25 μm^2^).

### Electroporation

Electroporation of neonatal mice of either sex was performed at P0 as previously described^52^. Electroporated retinas were harvested at P14. DNA constructs used for electroporation in this study are as follows: pCAG-Cre (Addgene plasmid 13775, deposited by C. Cepko^53^), pCALNL-GFP (Addgene plasmid 13770, deposited by C. Cepko^53^), pCAGIG-Nfia (Gateway cloned from Ultimate Human ORF Collection (Life Technologies)), pCAGIG-Plagl1 (Gateway cloned from Ultimate Human ORF Collection (Life Technologies)), pCAGIG-Rax (Gateway cloned from Ultimate Human ORF Collection (Life Technologies)), pCAGIG-Sox2 (Gateway cloned from Ultimate Human ORF Collection (Life Technologies)), pCAGIG-Sox8 (Gateway cloned from Ultimate Human ORF Collection (Life Technologies)), pCAGIG-Sox9 (Gateway cloned from Ultimate Human ORF Collection (Life Technologies)), pCAGGS-N1ICD (Addgene plasmid 26891, deposited by N. Gaiano^54^). All plasmids activate expression of their respective genes of interest, Cre recombinase, or GFP from the broadly active CAG promoter/enhancer.

### Immunohistochemistry

Fluorescent immunohistochemistry was performed on cryosectioned tissue as previously described^51^. Antibodies utilized for fluorescent immunohistochemistry are as follows: goat anti-GFP (1:500; Rockland Immunochemicals, Limerick, PA), rabbit anti-GFP (1:1000; Invitrogen, Waltham, MA), mouse anti-Glutamine synthase (GLUL) (1:200; BD Biosciences, San Jose, CA), mouse anti-KI67 (1:200; BD Biosciences, San Jose, CA), rabbit anti-LHX2 (1:1500; generated in house with Covance, Princeton, NJ), mouse anti-NFIA (1:200; CDI Laboratories), mouse anti-P27^Kip1^ (1:200; Invitrogen), mouse anti-PAX6 (1:200; Developmental Studies Hybridoma Bank, University of Iowa, Iowa City, IA), rabbit anti-phosphohistone H3 (PHH3) (1:200; Millipore, Billerica, MA). Secondary antibodies used were as follows: AlexaFluor488 conjugated donkey anti-goat IgG (1:500; Jackson Immunoresearch, West Grove, PA), AlexaFluor488 conjugated donkey anti-rabbit IgG (1:500; Jackson Immunoresearch, West Grove, PA), AlexaFluor594 conjugated donkey anti-rabbit IgG (1:500; Jackson Immunoresearch, West Grove, PA), AlexaFluor594 conjugated donkey anti-mouse IgG (1:500; Jackson Immunoresearch, West Grove, PA). All section immunohistochemical data shown was imaged and photographed on a Zeiss Meta 510 LSM confocal microscope.

### *In Situ* Hybridization

Single color *in situ* hybridization was performed as previously described^31^. The RNA probe for *Plagl1* was generated from the following EST sequence: GenBank accession number BE995637.

## Additional Information

**How to cite this article**: de Melo, J. *et al*. Multiple intrinsic factors act in concert with Lhx2 to direct retinal gliogenesis. *Sci. Rep.* **6**, 32757; doi: 10.1038/srep32757 (2016).

## Supplementary Material

Supplementary Information

## Figures and Tables

**Table 1:** Differential regulation of MG differentiation following overexpression of individual transcriptional regulators.

Gene	Wildtype			*Lhx2*-deficient		
	Radial	P27^Kip1^	GLUL	Radial	P27^Kip1^	GLUL
*Hes5*	+	+	+	0	0	0
*N1ICD*	+	−	−	−	+	+
*Nfia*	+	0	0	0	+	0
*Rax*	−	−	−	0	0	0
*Plagl1*	−	−	−	−	+	0
*Sox2*	0	0	0	−	0	0
*Sox8*	0	0	0	−	0	0
*Sox9*	0	0	−	−	+	0

Changes in the fraction of radial cells and both P27^Kip1^ and GLUL-positive following overexpression of the indicated factor are shown in both wildtype and *Lhx2*-deficient backgrounds. Increases (+), decreases (−), or unchanged (0) fractions of cells are relative to *Cre/GFP* controls in the indicated genetic background. *Hes5* data for P27^Kip1^ and GLUL is from ref. 21.
