# Supplementary material for: Multiple intrinsic factors act in concert with Lhx2 to direct retinal gliogenesis
Source: Sci Rep. 2016 Sep 8;6:32757. doi: 10.1038/srep32757 (PMC5015061; doi:10.1038/srep32757)
Supplement: Supplementary Information [file srep32757-s1.pdf]

Supplemental Information for the following manuscript:

Title: Multiple intrinsic factors act in concert with Lhx2 to direct retinal gliogenesis

Authors: Jimmy de Melo[1], Brian S. Clark[1], and Seth Blackshaw[1-5, *].
[1]Johns Hopkins University School of Medicine, Solomon H. Snyder Department of Neuroscience, Baltimore, 21205, USA
[2] Johns Hopkins University School of Medicine, Department of Ophthalmology, Baltimore, 21205, USA
[3] Johns Hopkins University School of Medicine, Department of Neurology, Baltimore, 21205, USA
[4] Johns Hopkins University School of Medicine, Center for Human Systems Biology, Baltimore, 21205, USA
[5] Johns Hopkins University School of Medicine, Institute for Cell Engineering, Baltimore, 21205, USA
*corresponding author (email: sblack@jhmi.edu)

Supplemental Table 1: Total cells counted for each gene and genetic background analyzed.

	Wildtype			*Lhx2*-deficient		
**Gene**	**Radial-AC**	**$P27^{Kip1}$**	**GLUL**	**Radial-AC**	**$P27^{Kip1}$**	**GLUL**
Cre	6064	2189	2456	4846	2022	1896
N1ICD	3290	1569	1410	3187	1530	1293
Nfia	4499	2190	2258	4295	2535	2591
Rax	5937	2867	2912	4244	3222	2885
Plagl1	6820	2879	2893	4286	2367	2511
Sox2	4766	2226	2393	5738	2634	2574
Sox8	5947	2807	2971	6923	3672	3465
Sox9	5617	2949	2642	5805	2888	2845

The total number of electroporated GFP labeled cells counted for each condition represents the sum of counts from 6 ($P27^{Kip1}$ and GLUL counts) or 12 (Radial and AC counts) independent electroporation replicates. AC=amacrine cell.

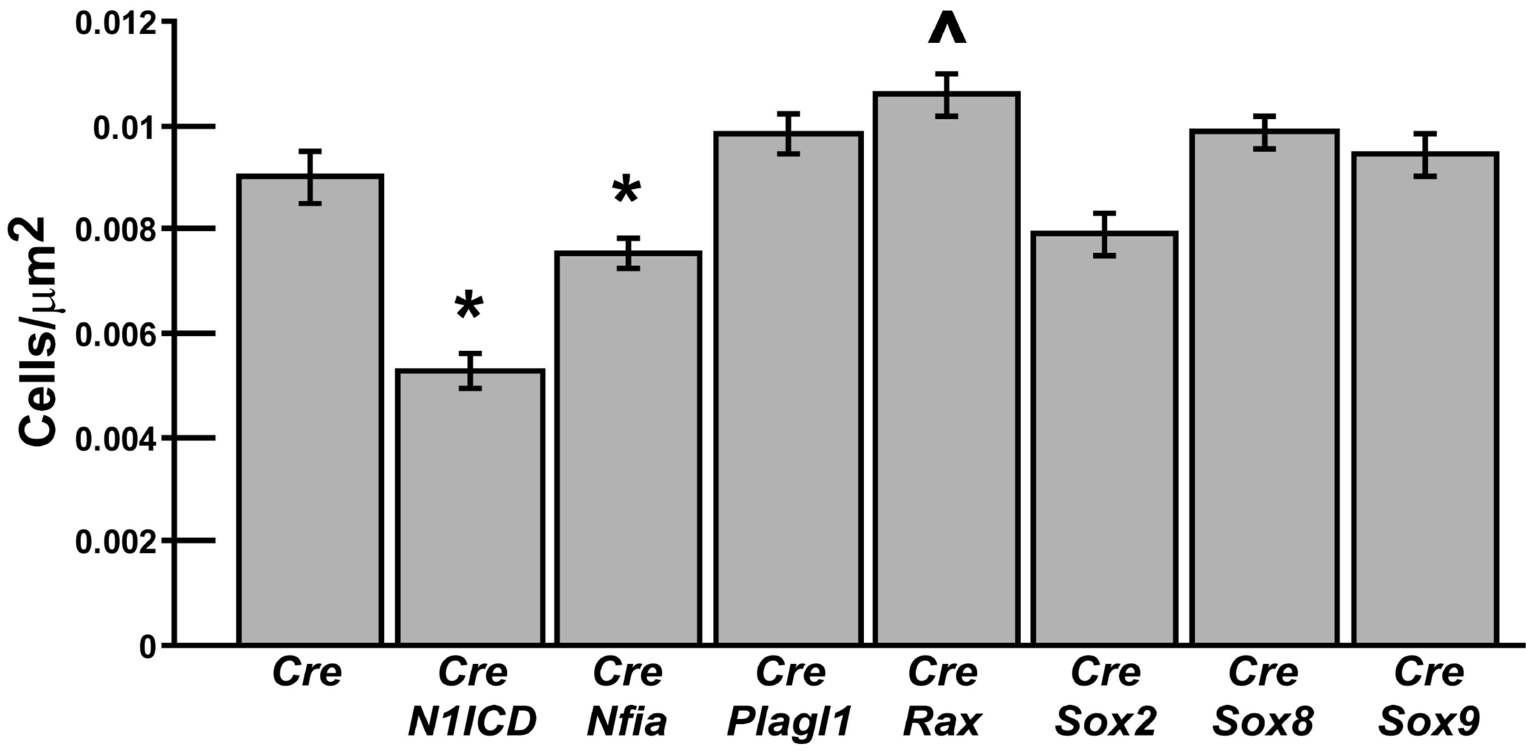

0.012
0.01
0.008
0.006
0.004
0.002
0
Cells/μm2
^
*
*
Cre
Cre N1ICD
Cre Nfia
Cre Plagl1
Cre Rax
Cre Sox2
Cre Sox8
Cre Sox9

Supplemental Figure 1. Quantification of density of electroporated cells/µm^2 in *Lhx2*$^{+/+}$ mice. In retinas electroporated with *Cre/GFP*, *Cre/GFP/N1ICD, Cre/GFP/Nfia, Cre/GFP/Plagl1, Cre/GFP/Rax, Cre/GFP/Sox2, Cre/GFP/Sox8, or Cre/GFP/Sox9* the cell density of electroporated cells was expressed as the number of GFP labeled cells per µm^2, where area equals the imaging field taken by the Zeiss Meta 510 LSM confocal microscope at 40X magnification. An asterisk (*) indicates a significant decrease in density of electroporated cells. A caret (^) indicates a significant increase in the cell density of the electroporated cells.
